# Clinical Ethics Consultation in Neurology – a case series

**DOI:** 10.1186/s12883-021-02244-2

**Published:** 2021-06-04

**Authors:** Benjamin Ilse, Bernd Alt-Epping, Albrecht Günther, Jan Liman, Alfred Simon

**Affiliations:** 1 Hans Berger Department of Neurology, Jena University Hospital, Jena, Germany; 2 Department of Palliative Medicine, University Hospital Heidelberg, Heidelberg, Germany; 3 Department of Neurology, University Medical Center Goettingen, Goettingen, Germany; 4 Academy of Ethics in Medicine, University Medical Center Goettingen, Goettingen, Germany

**Keywords:** Clinical decision-making, Clinical ethics consultation (CEC), Neurology, Individual patient will

## Abstract

**Background:**

The concept of clinical ethics consultation (CECs) was implemented to provide support in ethical controversies in clinical settings and are offered in at least every second hospital in Germany. Neurological disorders often require complex decision-making. The aims of this study were to determine which situations lead to CEC in neurology and to investigate the influence of the individual patient’s wishes on the recommendation.

**Methods:**

Standardised CEC protocols in the years 2011 to 2017 at the University Hospitals of Goettingen and Jena were retrospectively surveyed. The contents were categorised along existing protocol templates of CEC scenarios and subsequently paraphrased and reduced to significant meanings.

**Results:**

27 CEC scenarios which were facilitated by various professional disciplines were reviewed. Stroke was the most frequent underlying condition. Nearly all patients were not able to consent. Mostly, the relatives acted as representatives or health advocates. In 67 % of cases, a sense of conflict triggered a CEC; in 33 % a sense of uncertainty was the reason for the CEC request. In 21 CEC scenarios, a recommendation was reached in consensus with all parties involved. In 59 % of cases, a decision was made to continue medical therapy. In seven cases, the patient’s wishes led to a limitation of therapy, while in just two cases this decision was made primarily relying on the patient’s best interest. In only 13 % of cases, a valid advance directive led to respective therapeutic consequences.

**Conclusions:**

CEC is feasible for consensus-finding not only in conflicts, but also in situations of therapeutic uncertainty in neurology. There is a special importance of the patient’s wishes in decision-making in neurology. However, only in a few cases were advance directives precise and specific enough to have sufficient and decisive weight in therapeutic decision-making.

## Background

Clinical decision-making may result in ethically controversial and conflicting situations, especially when forgoing therapeutic interventions is being discussed [1]. This, in turn, may lead to moral distress [2]. Clinical ethics consultation (CEC) was implemented during the last two decades in order to provide support and counselling in ethical controversies in clinical settings [3, 4]. CEC demonstrated positive effects on satisfaction of participants and on health-related outcome parameters [5, 6]. To date, however, there is no systematic review or meta-analysis of controlled studies on the effectiveness of CEC [7], and previous attempts to evaluate CEC effectiveness focused rather on variables that reflect the process than on individual outcome parameters [8]. In Germany, CEC has been available since the end of the 1990s, and the number of such services in hospitals has been increasing continuously [9]. At present, ethics consultation or other ethical support services are offered in at least every second hospital, usually in the form of a health care ethics committee (HEC) [10].

CEC may be of particular importance in neurology for several reasons. The symptoms of patients with neurological disorders are often complex and diverse [11]. The characteristic courses of disease are often long and frequently complicated by behavioural changes, cognitive impairment and progressive speech and language disorders. This may possibly lead to an increased use of CEC. So far, one study from the US has been published on CEC in neurology [12]. For Germany, corresponding data are missing.

The objectives of the present study were to determine which situations lead to CEC in neurology and to investigate the influence of individual patient wishes (in the following referred to as “patient’s will” in accordance with German law) on the counselling recommendation.

## Methods

The study analyses the CECs, which were conducted in the years 2011 to 2017 on the neurological wards at the University Hospitals of Goettingen (UMG) and Jena (UKJ). The analysis is based on the protocols (or documentations) that were prepared of each CEC and became part of the patients’ files. The study was approved by the local institutional review boards (Jena Reg. No.: 2018-1096-Data, and Goettingen Application No.: 5/9/18An).

 The CECs took the form of facilitated ethical case deliberations on the wards. The facilitators were members of the respective HEC. They came from different professions (e.g. medical ethicists, physicians, nurses, and clergy) and were trained as ethics consultants according to the standards of the German Academy of Ethics in Medicine (AEM) [13]. CECs are performed when requested by patients, relatives, employees of the facilities, or other involved people. According to the “Standards for Ethics Consultation” [14], consensus is to be sought in CECs. Currently, the UMG has a capacity of approximately 1500 beds. The HEC of the UMG exists since 2010. From 2011 to 2017, there were a total of 145 inquiries and 78 ethical case discussions. Currently, there are approximately 1400 beds available at the UKJ. The HEC at the UKJ exists since 2004, with 75 inquiries and 68 ethical case discussions from 2011 to 2017 (missing data in 2013 and 2016). In addition to ethical case consultation, the HECs have responsibilities in ethical training and preparation of guidelines/recommendations.

To structure the CEC, a protocol template is used with the following content: problem (i.e. reason for the CEC); relevant medical, nursing and psycho-social facts; ethical evaluation; decision and further procedure. At the end of each CEC, a standardised protocol is prepared according to a predefined standard that contains the result and its justification and becomes part of the patient’s file.

The qualitative data analysis was carried out according to the content analytic flow model according to Mayring [15]. This included an initial evaluative category formation guided by the objectives of the study and along the present structure of the protocol templates used. A paraphrasing of the relevant sections from each standardised protocol followed this. In the next step, a generalisation to an abstraction level and a reduction were carried out. After coding via a code book, the evaluation with assignment to the evaluation categories proceeded inductively or, in one case, deductively. Evaluative categories were developed along the existing protocol templates of CEC scenarios. These were: the underlying disease, the ability to consent, the patient representative, the trigger event, the “seriousness” of this event, the form of expression of will, the ethical justification and the significance of the living will, and the result of the consultation. The existing contents of the standardised protocols were transferred to an Excel chart (Microsoft Excel). All data were pseudonymised. Access to personal data was restricted to employees of the respective institutions. Accordingly, the overall evaluation was carried out anonymously. In the evaluation categories mentioned, the contents of the standardised protocols were paraphrased and reduced to significant meanings with the formation of categories. A deductive category formation was carried out in the evaluation category “mode of expression of the patient’s will”. The following categories were used for this purpose: “currently declared will”, “previously declared will”, “presumed will”, “natural will”, and “no evidence of the patient’s will” [16]. Table 1 explains the different categories. All other evaluation categories were categorised inductively.

**Table 1:** mode of expression of the patient’s will

Patient’s will	Explanation
Currently declared will	the will of a patient who is currently capable of giving consent
Previously declared will	the will of a patient who is currently incapable of giving consent, as documented in a living will
Presumed will	individual presumed will of a patient who is currently incapable of giving consent, as assumed from concrete evidence
Natural will	current expression of will of a patient who is currently incapable of giving consent
No evidence of the patient’s will	

The content-analytical evaluation was carried out in each case by a member of the HEC. For data protection reasons, no exchange of raw data between the two institutions was allowed. After the data had been reduced, a meeting was held to check the plausibility of the category assignments made. Two representatives of each institution were present at this meeting. All methods were carried out in accordance with relevant guidelines and regulations.

## Results

### Patient characteristics

24 CEC cases and three follow-up consultations were reviewed (total: 27 CEC scenarios). The average age of the patients was 74 years (min 39, max 89 years). An average of five persons participated in the case discussion. The consultations were facilitated by two members of the HEC, on average, from various professional disciplines: medical ethicists, physicians, nurses, and clergy were involved.

In 16 of the 24 neurological patients, a stroke was the underlying condition (67 %). Both ischemic and haemorrhagic strokes were present. Other underlying conditions were (acute inflammatory) polyneuropathies (*n* = 2), infectious or autoimmune encephalitis (*n* = 2), status epilepticus (*n* = 2), and one case each of sepsis with posterior reversible encephalopathy syndrome (PRES) and hypoxia. At the time of the consultation, 13 of the patients were on ventilatory therapy. In retrospect, this information could not be reliably inferred from the standardised CEC protocols in four other patients.

In 22 of 24 cases, patients were not able to consent. In five cases, “aphasia” was listed as the cause of the lack of capacity to consent; in another five cases, it was attributed to “insufficient understanding” (e.g., dementia). In six other cases, “insufficient awareness” was listed as the cause of the lack of capacity to consent. “Ventilation with sedation” was explicitly listed as a cause in six other cases. In one case, the ability to consent was questionable. Only one patient with locked-in syndrome was reliably able to consent. In 14 patients, a relative acted as the patient’s representative (proxy or custodian). In one case, there was no patient’s representative; the courts were not engaged due to the clearly inferred medical prognosis.

### Reasons for HEC involvement (Table 2)

**Table 2:** Reasons for HEC involvement

Reasons		*n* = 27	
Uncertainty	team uncertain of whether measures were indicated	3	8
	team as well as patient representatives uncertain of whether measures were indicated or presumed to be wanted by the patient	3	
	patient representatives uncertain of whether measures were presumed to be wanted by the patient	2	
Conflict	disagreement among authorised relatives about the presumed will of the patient	1	16
	patient representatives rejected a proposed treatment option	11	
	patient representatives requested a questionable or non-indicated intervention	4	
	follow-up consultation	3	

In 16 of 24 cases (67 %), a conflict triggered a CEC. In 11 of these cases, the patient representatives rejected a proposed treatment option. In four cases, the patient representatives requested a questionable or non-indicated intervention. In one case, there was disagreement among authorised relatives about the presumed will of the patient.

In 8 cases (33 %), uncertainty (instead of an overt conflict) was the reason for the CEC request. These uncertainties concerned both the question of whether measures were indicated and whether measures were presumed to be wanted by the patient, and affected the treatment team as well as patient representatives. There was uncertainty whether to start early rehabilitation (2 x), whether a tracheostomy should be performed (2 x) or a pacemaker implanted, whether hospital treatment should be pursued, and whether the treatment goal should be rather symptom-guided and palliative (2 x).

For three patients, a follow-up consultation was planned and performed (two cases regarding tracheostomy and weaning, one case regarding PEG tube insertion).

### Results of ethical consultations (Table 3)

**Table 3:** Results of ethical consultations

	Recommendation	Supplement	*n* = 27	
Consensus	carry out or continue the therapy		2	21
		no recommendation for emergency arrangements	1	
		limiting medical therapy via recommendation for emergency arrangements	2	
		accompanied by a time limit recommendation for re-evaluation	2	
		accompanied by a time limit recommendation for re-evaluation; limiting medical therapy via recommendation for emergency arrangements	6	
	limiting medical therapy		7	
	recommending different scenarios for proceeding with the therapy		1	
No consensus	carry out or continue the therapy		1	6
		limiting medical therapy via recommendation for emergency arrangements	1	
		accompanied by a time limit recommendation for re-evaluation; limiting medical therapy via recommendation for emergency arrangements, no therapy intensification	1	
	limiting medical therapy		1	
	no therapeutic proposal was made	postponing the decision	2	
		legal clarification by court		

In 21 of 27 CEC scenarios, a recommendation for advice was achieved in consensus with all parties involved. In thirteen consultations, a recommendation was made to carry out or continue the therapy. In some of those, this recommendation was accompanied by a time limit, a recommendation for re-evaluation, or emergency arrangements. In seven consultations, a consensus was reached in favour of limiting medical therapy. One consultation discussed the therapeutic implications of different prognostic scenarios, instead of making an unequivocal recommendation.

In six CEC scenarios, no consensus was reached. In three of those, the initiation or continuation of a medical therapy was suggested, and once, forgoing therapy was recommended. In two cases, no therapeutic proposal was made at all. In one of these cases, the patient’s ability to consent was questionable. Here, a psychiatric consultation and legal clarification by a court was recommended.

### Ethical reasoning in consensus finding (Table 4)

**Table 4:** Ethical reasoning in consensus finding

CEC recommendation was primarily oriented towards…	Therapy	The patient’s will	*n* = 27	
… the patient’s will	positive and consenting will as decisive for the implementation or continuation of a medical therapy	expressed by the patient himself/herself	1	11
		assumed to be his/her presumed will	2	
		natural will	1	
	patient’s will lead to a limitation of therapy	"living will"	4	
		assumed to be his/her presumed will	3	
… the patient’s best interest	implementation or continuation would serve the patient’s best interest		1	10
		presumed will does not exclude this therapy	3	
		realistic chance that the patient’s health situation would improve, enabling him/her to decide	1	
		presumed consent	3	
	implementation or continuation would not serve the patient’s best interest		2	
Participants assessed differently whether the patient was able to consent			1	
Participants assessed differently what the patient’s will would be or what would be in his/her best interest			1	
Uncertainty among the treating physicians regarding the prognosis			4	

We examined whether the CEC recommendation was primarily oriented towards the will of the patient or the best interest of the patient (upon values deemed to be universally shared [17]). We could assume that the CEC recommendations were in the patient’s best interest, since the goal of CEC deliberations is to align decision-making processes with morally acceptable criteria [14].

In four cases, a positive and consenting will of the patient was identified as decisive for the implementation or continuation of a medical therapy. The will was both expressed by the patient himself/herself or was assumed to be his/her presumed or natural will. In seven cases, the patient’s will (either through a living will or as an individual presumed will) led to a limitation of therapy.

In eight cases, the idea that the implementation or continuation would serve the patient’s best interest was decisive for the CEC recommendation. In two cases, a decision to limit therapy was made primarily relying on the patient’s best interest.

In only two CEC scenarios did the participants differently assess whether the patient was able to consent, what his will would be or what would be in his/her best interest. In four cases, there was great uncertainty among the treating physicians regarding the prognosis, which, in turn, impeded the determination of the presumed patient’s will. In three of these cases, the continuation of the medical therapy was recommended until the prognosis was better assessed. In one case, a realistic chance was seen that the patient would improve to such an extent that he/she could decide on the continuation of the medical therapy. In another case, two different options how to proceed were outlined that could have been discussed between the patient representatives and the therapeutic team later. Both options were well compatible with the patient’s will and best interest, in the view of the ethics committee.

### Advance directive

In 14 of the 24 cases (58 %) no advance directive / living will was available. In five other cases (21 %) there was a living will, but these documents clearly did not apply to the current neurological situation. In another two cases (8 %), the living will was not clear or led to divergent assessments. In only three of 24 cases, a valid advance directive led to respective therapeutic consequences (13 %), another one was implemented after a follow-up consultation (where a former version that did not sufficiently apply to the current situation was adopted).

### Cases without consensus

In four of 24 cases a therapy recommendation by CEC was made without a consensus being reached. In none of these consultations was an advance directive available. The occasion for the ethics consultation was twice that the patient representatives rejected a proposed measure and twice that the patient representatives wanted a questionably indicated measure. A consensus could not be reached in two cases, as the patient representatives refused to participate in the consultation, referring to a disagreement about the presumed intention. In other cases, the patient representatives were overwhelmed and did not understand the actual situation, or the patient representatives could not see the reality and doubted the credibility of the prognosis.

### Implementation of the advisory recommendations

The question of whether the recommendations were implemented could methodically be investigated at only one study centre. After reviewing the concluding physician’s letters, the counselling recommendation was not implemented in one case out of 10. In half of the cases (five), the standardised CEC protocols became part of the patient files.

## Discussion

 To the best of our knowledge, the present study represents the first systematic evaluation of ethical counselling in neurology in German hospitals.

With 67 % of the patients who had a stroke as their main condition, the collective was comparable to a retrospective review on neurology patients in ethical case discussions from the US over seven years [12], in which stroke patients also prevailed. In inpatient neurology in Germany in general, though, stroke patients determine in median 23 % of all patients [18]. In these patients, severe impairments often result in long periods of hospitalisation on the one hand and existential issues on the other (such as continuation of nutritional therapy or respiratory therapy). Therefore, stroke patients are disproportionately often subject to ethical case discussions. This may probably be related to the resulting intensive care treatment, with a large spectrum of options to implement or to suspend therapeutic interventions on the one hand, and the acutely limited patient’s ability to consent on the other.

In 30 % of the described cases, the trigger for the consultation was not a conflict but an uncertainty (Table 2). Both the medical team and the patient representatives wanted to ensure the ethical appropriateness of a difficult decision by initiating a CEC. Patients with neurological disorders in particular often show a reduction in awareness and it is difficult to assess the prognosis [19]. This may determine the number of CEC scenarios due to uncertainty.

Fortunately, in the majority of cases (21 out of 27) a consensus recommendation could be made (Table 3). This can be interpreted as an indicator of efficacy of CEC, where participants and moderators seek consensus or, at least, a common terminology and mode of action. When no consensus was reached, a therapeutic limitation was recommended only once. In that case, intensive care was considered not to be indicated because of a dismal prognosis.

In the majority of cases (59 %, 16 out of 27), a decision was made to continue medical therapy. This decision often implied temporary therapeutic attempts and subsequent re-evaluations. This, in turn, was probably related to a lack of clarity on the patient’s will, as advance directives were not applicable to the current situation, or were missing altogether, and the patient was not able to participate. Another reason for the high number of pro-active decisions may be found in the difficult prognosis-finding in patients with severe acute neurological diseases. However, emergency arrangements were often made even in these cases. These arrangements usually included “do not resuscitate” or “do not (re-)intubate” orders. They were probably established with the idea in mind that further clinical deterioration and associated disability such as necessary long-term ventilation would presumably not be acceptable for the patient.

As an ethical justification for the result of the consultation, an orientation towards the patient’s will and the patient’s best interest were approximately equally frequent. If the will of the patient was known and was decisive for the “ethical justification”, refraining from further therapy was recommended more often than in cases where the decision was oriented merely towards the patient’s best interest. When the presumed will of the patient was not sufficiently known and therefore aspects of the patient’s best interest gained further importance, then recommendations tended to be made in favour of continuing the therapy (assuming, of course, the intervention was indicated). This may suggest that for a CEC recommendation to limit further therapeutic efforts in neurology, knowing the patient’s will is of high importance.

In only four cases, an advance directive determined the outcome of the consultation. This relatively small impact of advance directives also corresponds to a single centre evaluation in which living wills or advance directives were not associated with earlier withdrawal or withholding of life-sustaining therapy or therapeutic intensity on a neuro-intensive care unit [20].

## Conclusions

Our study demonstrates that CEC is feasible for consensus-finding in ethically conflicting situations in neurology. CEC is requested not only in conflicts, but also in situations of therapeutic uncertainty that may be caused by the special course of severe neurological disorders, where estimation of prognosis and communication remains difficult. The data presented also show the special importance of the patient’s will to accept therapy limitation decisions in neurology. However, only in a few cases were advance directives precise and specific enough to gain sufficient information on the patient’s will. Therefore, Advance Care Planning (ACP [21]) as a comprehensive, structured and facilitated conversation and counselling process that enables individuals to define and record goals and preferences for future medical treatment and care, could be helpful for patients with pre-existing or at risk of developing neurological disorders, as the usefulness of ACP has been already demonstrated for patients with Parkinson’s disease [22] or severe and progressive multiple sclerosis [23].

## Data Availability

The datasets generated and analysed during the current study are not publicly available due to data protection of the patients and their relatives but are available from the corresponding author on reasonable request.

## Abbreviations

ACP: Advance Care Planning
AEM: Academy of Ethics in Medicine
CEC: Clinical ethics consultation
DNI: Do not (re-)intubate
DNR: Do not resuscitate
HEC: Health Care Ethics Committee
ICU: Intensive care unit
IMC: Intermediate care unit
PEG: Percutaneous endoscopic gastrostomy
PRES: Posterior reversible encephalopathy syndrome
UMG: University Medical Center Goettingen
UKJ: Jena University Hospital
